# Supplementary material for: Estimating probabilities of malaria importation in southern Mozambique through modelling P. falciparum genomics and mobility patterns
Source: eLife. 2026 Jun 26;14:RP107136. doi: 10.7554/eLife.107136 (PMC13309124; doi:10.7554/eLife.107136)
Supplement: Peer review history

# Editor report: eLife Assessment

**DOI:** 10.7554/eLife.107136.4.sa0

**Authors:** Marcelo U Ferreira

**Affiliations:** University of São Paulo Brazil

**Keywords:** Convincing, Important

This study introduces an **important** method to estimate the probability of malaria importation with a new Bayesian approach that integrates epidemiological, travel reports, and genetic data. The authors provide **convincing** evidence for the value of this model in identifying the main sources of malaria transmission and risk factors. This work will be of interest to the area of genomic epidemiology and public health strategies aiming to eliminate malaria.

# Referee report: Reviewer #1 (Public review)

**DOI:** 10.7554/eLife.107136.4.sa1

Summary:

This study presents a new Bayesian approach to estimate importation probabilities of malaria combining epidemiological data, travel history, and genetic data through pairwise IBD estimates. Importation is an important factor challenging malaria elimination, especially in low transmission settings. This paper focus on Magude and Matutuine, two districts in south Mozambique with very low malaria transmission. The results show isolation-by-distance in Mozambique, with genetic relatedness decreasing with distances larger than 100 km, and no spatial correlation for distances between 10 and 100 km. But again strong spatial correlation in distances smaller than 10 km. They report high genetic relatedness between Matutuine and Inhambane, higher than between Matutuine and Magude. Inhambane is the main source of importation in Matutuine, accounting for 63.5% of imported cases. Magude, on the other hand, shows smaller importation and travel rates than Matutuine, as it is a rural area with less mobility. Additionally, they report higher levels of importation and travel in the dry season, when transmission is lower. Also, no association with importation was found for occupation, sex and other factors. These data have practical implications for public health strategies aiming malaria elimination, for example, testing and treating travelers from Matutuine in the dry season.

Strengths:

The strength of this study relies in the combination of different sources of data - epidemiological, travel and genetic data - to estimate importation probabilities, the statistical analyses.

Weaknesses:

The authors recognize the limitations related to sample size and the biases of travel reports.

# Referee report: Reviewer #2 (Public review)

**DOI:** 10.7554/eLife.107136.4.sa2

Summary:

Based on a detailed dataset, the authors present a novel Bayesian approach to classify malaria cases as either imported or locally acquired.

Strengths:

The proposed Bayesian approach for case classification is simple, well justified, and allows the integration of parasite genomics, travel history, and epidemiological data.

Weakness:

While the authors aim to classify cases as imported or locally acquired, the method does not quantify the contribution of each case type to overall transmission, which the authors leave for future study.

# Referee report: Reviewer #3 (Public review)

**DOI:** 10.7554/eLife.107136.4.sa3

This work provides a novel statistical model to identify imported malaria cases, which are an important challenge for elimination, particularly in low-transmission areas. This tool was applied in *Plasmodium falciparum* populations in Mozambique and determined differences in importation rates in two low-transmission districts in the South.

Strengths:

The study has several strengths, particularly the development of a novel Bayesian model integrating genomic, epidemiological, and travel data to estimate importation probabilities. The findings provided important insights into malaria transmission dynamics, including the identification of importation sources and regional differences in importation rates across Mozambique. These results highlight the potential value of targeted interventions among traveler populations to support malaria elimination efforts. Moreover, this approach could be adapted to other epidemiological settings.

Weaknesses:

The study has some limitations, including uneven sample representation across provinces, incomplete metadata for risk factor analysis and a proxy for transmission intensity. Future work will include a new sample collection effort and the incorporation of monthly malaria incidence estimates.

# Author comment: Author response

**DOI:** 10.7554/eLife.107136.4.sa4

**Authors:** Arnau Pujol, Arlindo Chidimatembue, Clemente da Silva, Simone Boene, Henriques Mbeve, Pau Cisteró, Carla García-Fernández, Arnau Vañó-Boira, Dário Tembisse, José Inácio, Glória Matambisso, Fabião Luis, Nelo Ndimande, Humberto Munguambe, Lidia Nhamussua, Wilson Simone, Andrés Aranda-Díaz, Manuel García-Ulloa, Neide Canana, Maria Tusell, Júlia Montaña, Laura Fuente-Soro, Khalid Ussene Bapu, Maxwell Murphy, Bernardete Rafael, Eduard Rovira-Vallbona, Caterina Guinovart, Bryan Greenhouse, Sonia Maria Enosse, Francisco Saúte, Pedro Aide, Baltazar Candrinho, Alfredo Mayor

**Affiliations:** Barcelona Institute for Global Health Barcelona Spain; Manhiça Health Research Centre Manhiça Mozambique; Manhiça Health Research Centre Manhiça Mozambique; Manhiça Health Research Centre Manhiça Mozambique; Manhiça Health Research Centre Manhiça Mozambique; ISGlobal Barcelona Spain; Barcelona Institute for Global Health Barcelona Spain; Barcelona Institute for Global Health Barcelona Spain; Manhiça Health Research Centre Manhiça Mozambique; Manhiça Health Research Centre Manhiça Mozambique; Manhiça Health Research Centre Manhiça Mozambique; Manhiça Health Research Centre Manhiça Mozambique; Manhiça Health Research Centre Manhiça Mozambique; Manhiça Health Research Centre Manhiça Mozambique; Manhiça Health Research Centre Manhiça Mozambique; Manhiça Health Research Centre Manhiça Mozambique; Barcelona Institute for Global Health Barcelona Spain; Barcelona Institute for Global Health Barcelona Spain; Malaria Consortium Maputo Mozambique; Barcelona Institute for Global Health Barcelona Spain; Barcelona Institute for Global Health Barcelona Spain; ISGlobal Barcelona Spain; Manhiça Health Research Centre Manhiça Mozambique; University of California, San Francisco San Francisco United States; National Malaria Control Program Maputo Mozambique; Instituut voor Tropische Geneeskunde Antwerp Belgium; Barcelona Institute for Global Health Barcelona Spain; University of California, San Francisco San Francisco United States; Malaria Consortium Maputo Mozambique; Manhiça Health Research Centre Manhiça Mozambique; Manhiça Health Research Centre Manhiça Mozambique; National Malaria Control Program Maputo Mozambique; ISGlobal Barcelona Spain

The following is the authors’ response to the previous reviews

**Public Reviews:**

**Reviewer #1 (Public review):**

Summary:

This study presents a new Bayesian approach to estimate importation probabilities of malaria combining epidemiological data, travel history, and genetic data through pairwise IBD estimates. Importation is an important factor challenging malaria elimination, especially in low transmission settings. This paper focus on Magude and Matutuine, two districts in south Mozambique with very low malaria transmission. The results show isolation-by-distance in Mozambique, with genetic relatedness decreasing with distances larger than 100 km, and no spatial correlation for distances between 10 and 100 km. But again strong spatial correlation in distances smaller than 10 km. They report high genetic relatedness between Matutuine and Inhambane, higher than between Matutuine and Magude. Inhambane is the main source of importation in Matutuine, accounting for 63.5% of imported cases. Magude, on the other hand, shows smaller importation and travel rates than Matutuine, as it is a rural area with less mobility. Additionally, they report higher levels of importation and travel in the dry season, when transmission is lower. Also, no association with importation was found for occupation, sex and other factors. These data have practical implications for public health strategies aiming malaria elimination, for example, testing and treating travelers from Matutuine in the dry season.

Strengths:

The strength of this study relies in the combination of different sources of data - epidemiological, travel and genetic data - to estimate importation probabilities, the statistical analyses.

Weaknesses:

The authors recognize the limitations related to sample size and the biases of travel reports.

We appreciate the review and comment about the manuscript.

**Reviewer #2 (Public review):**

Summary:

Based on a detailed dataset, the authors present a novel Bayesian approach to classify malaria cases as either imported or locally acquired.

Strengths:

The proposed Bayesian approach for case classification is simple, well justified, and allows the integration of parasite genomics, travel history, and epidemiological data.

Weakness:

While the authors aim to classify cases as imported or locally acquired, the work lacks a quantification of the contribution of each case type to overall transmission.

Comments on revisions:

All my questions and concerns were satisfactorily addressed.

We appreciate the review and comment about the manuscript. In fact, the approach does not pretend to quantify the contribution of each case to overall transmission. In the discussion we state it and refer to future work with this scope.

**Reviewer #3 (Public review):**

This work provides a novel statistical model to identify imported malaria cases, which are an important challenge for elimination, particularly in low-transmission areas. This tool was applied in *Plasmodium falciparum* populations in Mozambique and determined differences in importation rates in 2 low-transmission districts in the South.

Strengths:

The study has several strengths, mainly the development of a novel Bayesian model that integrates genomic, epidemiological, and travel data to estimate importation probabilities. The results showed insights into malaria transmission dynamics, particularly identifying importation sources and differences in importation rates in Mozambique. Finally, the relevance of the findings is to suggest interventions focusing on the traveler population to support efforts for malaria elimination.

Weaknesses:

The study also has some limitations, although the authors have plans to address them. The sample collection was not representative of some provinces, and not all samples had sufficient metadata for the risk factor analysis. Additionally, the authors used a proxy for transmission intensity and assumed some other conditions to calculate the importation probability for specific scenarios. They plan to conduct a new sample collection and include monthly malaria incidence estimates in the future.

Comments on revisions:

Delete "We added this text to the discussion" in line 302 (Discussion)

I recommend adding the plans to address limitations indicated in the Response to Reviewers document in the Discussion. This would really strengthen the limitation section.

Thank you for pointing to these aspects. We deleted the sentence mentioned. In the discussion section, we now finish the paragraph on limitations with the proposed future work to address them.
